# Pre-Clade IIb Mpox Virus Exposure in Ghana: A Retrospective Serological Analysis

**DOI:** 10.3390/v17111415

**Published:** 2025-10-24

**Authors:** Christopher Dorcoo, Grace Opoku Gyamfi, Franziska Kaiser, Elvis Suatey Lomotey, Jeffrey Gabriel Sumboh, Robert J. Fischer, Claude Kwe Yinda, Vincent J. Munster, Joseph H. K. Bonney, Irene Owusu Donkor

**Affiliations:** 1 Parasitology Department, Noguchi Memorial Institute for Medical Research, University of Ghana, Legon P.O. Box LG 581, Ghana; cdorcoo@noguchi.ug.edu.gh (C.D.); gogyamfi@noguchi.ug.edu.gh (G.O.G.); elomotey@noguchi.ug.edu.gh (E.S.L.); jsumboh@gmail.com (J.G.S.); 2 Virus Ecology Section, Laboratory of Virology, Rocky Mountain Laboratories, National Institute of Allergy and Infectious Diseases, National Institutes of Health, Hamilton, MT 59840, USA; franziska.kaiser@nih.gov (F.K.); fischer.robertjon@gmail.com (R.J.F.); yinda.kweclaude@nih.gov (C.K.Y.); vincent.munster@nih.gov (V.J.M.); 3 Virology Department, Noguchi Memorial Institute for Medical Research, University of Ghana, Legon P.O. Box LG 581, Ghana; kbonney@noguchi.ug.edu.gh; 4 Epidemiology Department, Noguchi Memorial Institute for Medical Research, University of Ghana, Legon P.O. Box LG 581, Ghana

**Keywords:** mpox, MPXV, seroprevalence, serosurvey, Ghana, risk factors

## Abstract

*Monkeypox virus* (MPXV), a zoonotic Orthopox virus endemic to West and Central Africa, causes mpox disease. Although Ghana had no confirmed human cases before 2022, the 2003 U.S. mpox outbreak was traced to rodents exported from Ghana, suggesting potential undetected exposure in the local population. This study assessed mpox exposure prior to the emergence of Clade IIb in humans. We tested 457 serum samples collected across 14 regions of Ghana using a commercial anti-MPXV IgG ELISA. These samples comprised 365 archived sera from 2021 SARS-CoV-2 surveillance and 92 sera from suspected mpox cases during the 2022 outbreak. Multivariable logistic regression was performed to examine associations between MPXV seropositivity and demographic factors, including age, sex, region, urban/rural status and inferred smallpox vaccination status. Overall MPXV seroprevalence was 6.6%. Participants from the Western Region had significantly increased odds of seropositivity (aOR = 6.70, 95% CI: 1.75–25.62, *p* = 0.005), whereas those from Greater Accra had decreased odds (aOR = 0.28, 95% CI: 0.09–0.90, *p* = 0.033). The findings suggest localized MPXV circulation or repeated zoonotic spillover may have occurred undetected, challenging the prevailing assumption that Ghana was unaffected by human mpox prior to 2022, underscoring the importance of strengthened surveillance and preparedness in Ghana.

## 1. Introduction

Mpox, caused by the *Monkeypox virus* (MPXV), is an emerging zoonotic disease of increasing global health concern. MPXV is an enveloped double-stranded DNA virus of the *Orthopoxvirus* genus [1]. Two distinct clades of *Monkeypox virus* (MPXV) are recognized: Clade I, historically circulating in Central Africa, and Clade II, originating in West Africa, with further subdivisions into lineages Ia/Ib and IIa/IIb, respectively [2,3]. While outbreaks were once sporadic and largely linked to zoonotic spillover, the past two decades have seen a steady rise in incidence and geographic expansion [4].

In May 2022, Clade IIb MPXV triggered an unprecedented multinational outbreak that rapidly spread across non-endemic regions, prompting the World Health Organization (WHO) to declare mpox a Public Health Emergency of International Concern (PHEIC) [5]. More recently, Clade Ib has fueled a resurgence of cases in the Democratic Republic of the Congo (DRC) and neighboring countries, including Uganda, Burundi, Rwanda, Kenya and Zambia [6]. with evidence of exportation beyond Africa—Sweden, India, Germany and the United States of America [7,8]. As of 26 January 2025, 21 countries have reported 19,837 confirmed cases, including 70 deaths [9]. Case Fatality Ratios (CFR) have been reported to differ substantially between clades, ranging from <1% in many clade II outbreaks to >10% in Clade I outbreaks, particularly in Central Africa [10,11,12].

Ghana represents a paradox in the epidemiology of mpox. Although the country had no confirmed human cases before 2022, it played a central role in the 2003 U.S. outbreak when rodents exported from Ghana seeded transmission in prairie dogs [13,14].

In 2022, Ghana reported its first human cases of mpox, interestingly in a male traveler returning from the United States of America with mild symptoms [15,16]. By the last quarter of the year, Ghana Health Service recorded a cumulative total of 87 suspected cases, of which 76 were laboratory-confirmed, with four associated deaths [15,17]. This has raised urgent questions about whether undetected transmission might have occurred locally before the recognition of Clade IIb spread. Given Ghana’s ecological diversity, bushmeat consumption, and wildlife trade links, silent MPXV circulation cannot be excluded.

To address this gap, we conducted a retrospective serological analysis of archived sera collected across Ghana prior to the 2022 outbreak to investigate evidence of undetected MPXV exposure. In addition, we analyzed clinical samples obtained during the 2022 outbreak to provide a contemporary comparator. Together, these datasets allowed us to evaluate both pre-outbreak exposure and outbreak-associated serological responses, thereby offering new insights into the hidden epidemiology of mpox in Ghana and informing regional preparedness in West Africa.

## 2. Methods

### 2.1. Study Design

This study employed a retrospective cross-sectional study approach using samples collected during a SARS-CoV-2 surveillance study conducted across 16 regions of Ghana. A total of 5939 serum samples were collected between February and December 2021 for this study [18]. Samples for this study were selected based on reported suspected and confirmed cases during the outbreak, availability of sufficient sample volume and completeness of demographic metadata. Clinical samples from suspected cases during the 2022 national mpox outbreak were screened for MPXV antibodies. All sample-related data were deidentified to ensure confidentiality.

### 2.2. Study Population and Sample Size

**Figure 1:**
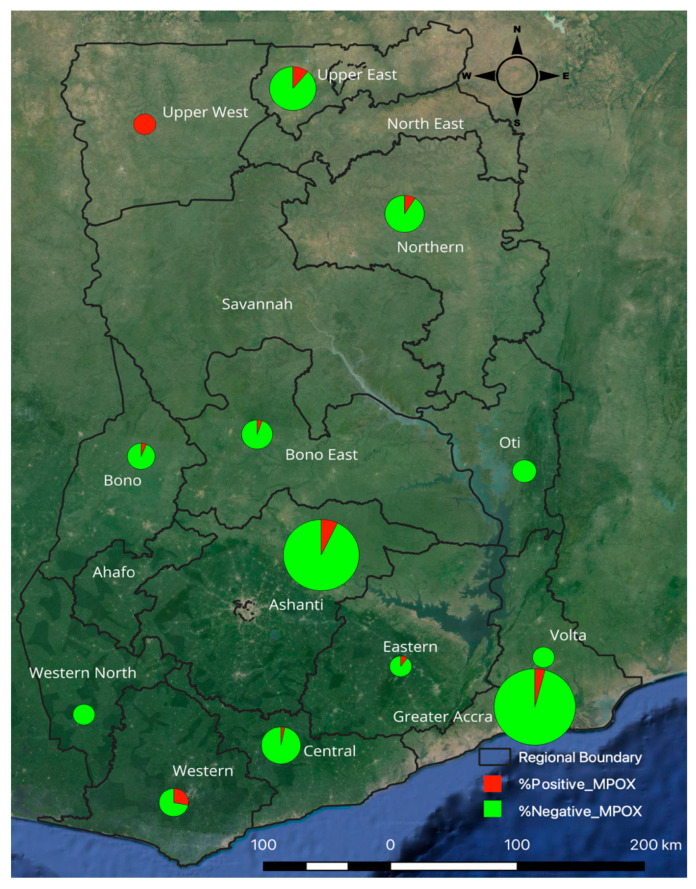
Regional distribution of MPXV seroprevalence in Ghana. Circle diameters represent the total number of samples collected in each region.

This study analyzed a total of 457 serum samples representing 14 regions of Ghana. A subset of 365 archived sera collected during SARS-CoV-2 surveillance activities in 2021 [18] and 92 clinical sera obtained from individuals during the 2022 national outbreak [19]; however, data on clinical symptoms associated with the clinical samples collected during the 2022 national outbreak were unavailable. Samples were collected by venipuncture into Serum Separating Tubes (SSTs) by trained healthcare personnel following standard biosafety procedures. Immediately after collection, whole blood was processed to obtain serum, which was aliquoted into cryovials, stored at −20 °C at sentinel sites, and later transported on a cold chain to the Noguchi Memorial Institute for Medical Research (NMIMR), where they were preserved at −80 °C until analysis. To ensure broad geographic representation, archived samples were selected across all regions with emphasis on highly populated areas and regions with suspected or confirmed mpox cases, particularly Greater Accra and Ashanti, which are Ghana’s largest urban centers and major hubs of domestic and international travel (Figure 1).

### 2.3. Sample Processing

All samples were processed and analyzed at the Noguchi Memorial Institute for Medical Research and the Rocky Mountain Laboratories, NIAID, USA.

### 2.4. Detection of IgG Antibodies Against MPVX

All sera were screened for anti-MPXV IgG antibodies using a commercial ELISA kit (RayBiotech, Peachtree Corners, Peachtree Corners, GA, USA) targeting the MPXV E8L protein (RayBiotech, Peachtree Corners, GA, USA), following the manufacturer’s instructions. Serum samples were diluted 1:200 using the assay diluent provided in the RayBiotech ELISA kit (Catalog No. ELV-MPXVE8L), in accordance with the manufacturer’s instructions for serum/plasma samples. The diluted samples were then added to the pre-coated 96-well microplate and processed following the recommended protocol. Each 96-well plate included negative control sera from unexposed individuals (individuals with no known history of exposure to mpox or vaccination). The cut-off for seropositivity was defined as the mean optical density of the negative controls plus ten (10×) standard deviations. This ELISA has a sensitivity of 0.11 pg/mL and is designed with high specificity as antibody pair detects Monkeypox Virus (MPXV) Envelope protein E8L with no reported cross-reactivity to other orthopoxviruses [20].

### 2.5. Data Curation and Analysis

Sample metadata, including GPS location, demographic variables from surveillance records and clinical status, were collated using Microsoft excel. All records were reviewed for completeness, standardized, and de-identified prior to analysis to ensure confidentiality. Smallpox vaccination status was defined based on the existing criteria [21]. Briefly, individuals born before the official smallpox eradication declaration in 1980 (≥45 years) were classified as vaccinated. Those born between 1975 and 1985 (40–50 years) were considered to have an uncertain vaccination status, while individuals born after 1985 (≤39 years) were classified as unvaccinated. The finalized dataset was imported into Stata version 17 MP4 (StataCorp, College Station, TX, USA) for statistical analyses. Seroprevalence was estimated with corresponding 95% confidence intervals (CIs). Associations between MPXV seropositivity and demographic variables (age group, sex), geographic region, and smallpox vaccination status were assessed using multivariable logistic regression. Results were expressed as odds ratios (ORs) with 95% CIs, and statistical significance was defined as *p* < 0.05.

## 3. Results

A total of 457 serum samples were analyzed, comprising both archived sera and clinical specimens collected during the 2022 outbreak. Just under two-thirds of participants resided in urban areas (62.6%), while 17.3% were from rural settings. The age distribution was skewed toward younger individuals: more than half were ≤25 years, with nearly one quarter under 16 years. Females constituted a slight majority (55.4%). Regional representation was greatest from Greater Accra (30.0%) and Ashanti (26.0%), reflecting their high population density and role as major travel hubs. Vaccination status, inferred from birth year, indicated that two-thirds of participants were classified as unvaccinated against smallpox (67.8%), while only one in five were considered vaccinated (22.1%) (Table 1).

### Factors Associated with MPXV Seropositivity in the Tested Samples

In multivariable models, neither sex, age, nor smallpox vaccination status was significantly associated with MPXV seropositivity. Geographic factors, however, showed strong associations. Participants from the Western Region had significantly higher odds of seropositivity (aOR = 6.70, 95% CI: 1.75–25.62; *p* = 0.005), whereas those from Greater Accra had significantly lower odds (aOR = 0.28, 95% CI: 0.09–0.90; *p* = 0.033). Strikingly, clinical samples with undisclosed location data also showed elevated odds of seropositivity (aOR = 5.65, 95% CI: 1.52–20.94; *p* = 0.010) (Table 2).

## 4. Discussion

This retrospective serological study suggests nationwide evidence of prior mpox virus (MPXV) exposure in Ghana before the detection of Clade IIb in humans in 2022. We observed an overall seroprevalence of 6.6%, with signals of elevated exposure in the Western Region and among younger, largely unvaccinated individuals. These findings suggest that localized MPXV circulation or repeated zoonotic spillover may have occurred undetected, challenging the prevailing assumption that Ghana was unaffected by human mpox prior to 2022 [12]. Moreover, this interpretation lends further support to earlier evidence implicating Ghana as the source of the reservoirs linked to the 2003 outbreak in the United States [14].

The geographic divergency warrants particular attention as well as careful interpretation. While samples from the Western Region showed significantly increased odds of seropositivity compared to the Ashanti Region, Greater Accra showed reduced odds of seropositivity; these patterns may reflect both genuine epidemiological differences and/or potential biases arising from the sample constitution. Ecological interfaces, wildlife trade, and bushmeat consumption practices in the Western Region plausibly elevate zoonotic spillover, all of which modulate human mpox exposure risk [22,23], but the uneven distribution of archived samples across regions, coupled with purposive inclusion of areas considered high-risk, may have contributed to the apparent differences; hence, it should be regarded as exploratory and interpreted within the context of the study’s sampling limitations. Nonetheless, these findings align with ecological hypotheses of localized exposure and are consistent with current epidemiological patterns, wherein 71% of recent mpox cases were reported from the Western Region [24,25].

The Western Region is rich in tropical forest ecosystems with dense rodent and primate populations, potential reservoirs for orthopoxvirus [26,27]. On the other hand, Greater Accra, while a hub for international travel, has lower direct zoonotic exposure. Such patterns suggest the importance of integrating ecological and behavioral factors into mpox risk assessments across West Africa [28].

Our findings revealed that seropositivity was highest among individuals under 35 years, the cohort not covered by routine smallpox vaccination. This aligns with the broader African and global experience, where waning Orthopoxvirus immunity after the cessation of smallpox vaccination has widened the pool of susceptible hosts [29,30]. Although our analysis did not demonstrate a statistically significant protective effect of inferred smallpox vaccination status, likely due to limited sample size, the pattern is consistent with reports of increased mpox burden in younger, unvaccinated populations during the 2022 multinational outbreak [31].

Our findings also intersect with Ghana’s paradoxical role in mpox epidemiology. Despite the absence of confirmed human cases until 2022, Ghana was central to the 2003 U.S. outbreak via exported rodents [14]. The present serological evidence suggests that humans within Ghana may have experienced unrecognized exposures long before international recognition, highlighting the limitations of passive surveillance systems in detecting low-level or atypical infections. This echoes broader concerns that mpox epidemiology in Africa is incompletely understood, with the true burden underestimated by reliance on syndromic case detection, i.e., identifying cases based solely on clinical symptoms [10,32].

Several limitations merit consideration. First, serological assays for MPXV may cross-react with other orthopoxviruses, potentially inflating prevalence estimates. However, the use of the E8L-targeting ELISA provides enhanced specificity. Second, the modest sample size, particularly within certain regions, limited statistical power to detect finer-scale associations. Third, the lack of confirmatory assays like seroneutralization, PCR and/or viral sequencing constrains definitive attribution of exposure to MPXV versus related viruses. Finally, the absence of detailed information on the stage of illness at sample collection may have influenced antibody detection and interpretation of serological patterns. Nonetheless, the consistency of our findings with known ecological and epidemiological patterns lends credibility to the observed patterns. Moreover, future studies should address these limitations by integrating molecular and serological approaches, including IgM/IgG detection, PCR and neutralization assays; expanding sample size and geographic coverage; and collecting detailed clinical, ecological, behavioral and exposure data to enable more comprehensive interpretation of mpox transmission dynamics.

In conclusion, this study suggests that Ghanaians have likely been exposed to MPXV prior to the recognition of Clade IIb transmission, with implications for surveillance, preparedness, and risk communication. Strengthened One Health-based surveillance that integrates human, animal, and ecological data is essential to detect early zoonotic transmission events. Regional and global health security strategies must also recognize that the epidemiology of mpox in West Africa extends beyond recognized outbreaks. Future research should prioritize longitudinal serological studies, reservoir host investigations, and genomic surveillance to better define the landscape of MPXV circulation and mitigate the risk of future widespread outbreaks.

## Figures and Tables

**Table 1:** Sociodemographic characteristics of samples tested.

Characteristic	Frequency (N = 457)	Percentage (%)
Area		
Rural	79	17.29
Urban	286	62.58
Age		
<16	114	24.95
16–25	127	27.79
26–35	65	14.22
36–45	50	10.94
46–55	47	10.28
56–65	29	6.35
65+	25	5.47
Sex		
Female	253	55.36
Male	204	44.64
Region		
Ashanti	119	26.04
Bono East	20	4.38
Brong Ahafo	19	4.16
Central	32	7.00
Eastern	10	2.19
Greater Accra	137	29.98
Northern	32	7.00
Oti	12	2.63
Upper East	45	9.85
Upper West	1	0.22
Volta	9	1.97
Western	18	3.94
Western North	3	0.66
Vaccination Status		
Unvaccinated	310	67.83
Vaccinated	101	22.1
Uncertain vaccination status	46	10.07

**Table 2:** Factors associated with MPXV seropositivity among the tested samples.

Characteristics	Seropositivity		cOR (95% CI)	*p*-Value	aOR (95% CI)	*p*-Value
	Negative	Positive				
Sex						
Female	238 (94.07)	15 (5.93)	Ref		Ref	
Male	189 (92.65)	15 (7.35)	1.259 (0.6–2.641)	0.542	1.158 (0.509–2.636)	0.726
Age (years)						
<16	102 (89.47)	12 (10.53)	Ref		Ref	
16–25	121 (95.28)	6 (4.72)	0.421 (0.153–1.163)	0.095	0.412 (0.135–1.259)	0.12
26–35	60 (92.31)	5 (7.69)	0.708 (0.238–2.109)	0.536	0.645 (0.203–2.048)	0.457
36–45	48 (96.00)	2 (4.00)	0.354 (0.076–1.645)	0.185	0.367 (0.065–2.087)	0.259
46–55	45 (95.74)	2 (4.26)	0.378 (0.081–1.758)	0.215	0.433 (0.086–2.171)	0.309
56–65	29 (100.00)	0 (0.00)	1		1	
65+	22 (88.00)	3 (12.00)	1.159 (0.302–4.455)	0.83	1.755 (0.416–7.406)	0.444
Location						
Rural	73 (92.41)	6 (7.59)	Ref		Ref	
Undisclosed	80 (86.96)	12 (13.04)	1.825 (0.652–5.112)	0.252	5.649 (1.524–20.943)	0.01
Urban	274 (95.80)	12 (4.20)	0.533 (0.193–1.468)	0.223	1.094 (0.337–3.55)	0.881
Region						
Ashanti	111 (93.28)	8 (6.72)	Ref		Ref	
Bono East	19 (95.00)	1 (5.00)	0.73 (0.086–6.176)	0.773	0.543 (0.051–5.844)	0.615
Brong Ahafo	18 (94.74)	1 (5.26)	0.771 (0.091–6.536)	0.811	1.1 (0.105–11.516)	0.936
Central	31 (96.88)	1 (3.13)	0.448 (0.054–3.716)	0.457	0.341 (0.051–2.304)	0.27
Eastern	9 (90.00)	1 (10.00)	1.542 (0.173–13.734)	0.698	1.625 (0.123–21.467)	0.712
Greater Accra	132 (96.35)	5 (3.65)	0.526 (0.167–1.652)	0.271	0.281 (0.088–0.903)	0.033
Northern	29 (90.63)	3 (9.38)	1.435 (0.358–5.754)	0.61	0.661 (0.132–3.324)	0.616
Oti	12 (100.00)	0 (0.00)	1		1	
Upper East	40 (88.89)	5 (11.11)	1.734 (0.536–5.613)	0.358	0.527 (0.137–2.026)	0.351
Upper West	1 (100.00)	0 (0.00)	1		1	
Volta	9 (100.00)	0 (0.00)	1		1	
Western	13 (72.22)	5 (27.78)	5.337 (1.519–18.746)	0.009	6.704 (1.754–25.618)	0.005
Western North	3 (100.00)	0 (0.00)	1		1	
Vaccination Status						
Uncertain	44 (95.65)	2 (4.35)	Ref		Ref	
Vaccinated	96 (95.05)	5 (4.95)	1.146 (0.214–6.137)	0.874	1.518 (0.238–9.693)	0.659
Unvaccinated	287 (92.58)	23 (7.42)	1.763 (0.402–7.74)	0.452	1.788 (0.304–10.52)	0.521

## Data Availability

All data generated or analyzed during this study are included in this published article.
